# Baseline AI Economic Literacy and Enacted Adoption of Artificial Intelligence Tools by Oncology and Critical Care Professionals

**DOI:** 10.3390/curroncol33100626

**Published:** 2026-10-16

**Authors:** Dragoș-Ciprian Negoiță, Ciprian Gindac, Akhil Akula, Karina Cristina Marin, Adrian Cosmin Ilie

**Affiliations:** 1 Doctoral School, Faculty of Medicine, “Victor Babeș” University of Medicine and Pharmacy Timișoara, 300041 Timișoara, Romania; dragos.negoita@umft.ro; 2 Anesthesiology and Intensive Care Department, “Victor Babeș” University of Medicine and Pharmacy Timișoara, 300041 Timișoara, Romania; ciprian.gindac@umft.ro; 3 Clinic of Anaesthesia and Intensive Care, “Pius Brînzeu” Emergency County Hospital, 300723 Timișoara, Romania; 4 Anaesthesia and Intensive Care Research Center (CCATITM), Faculty of Medicine, “Victor Babeș” University of Medicine and Pharmacy Timișoara, 300041 Timișoara, Romania; 5 Kaloji Narayana Rao University of Health Sciences, Warangal 506007, India; akhil.akula1998@gmail.com; 6 Department of Otorhinolaryngology, Faculty of Medicine, “Victor Babeș” University of Medicine and Pharmacy Timișoara, 300041 Timișoara, Romania; 7 Discipline of Public Health, Center for Translational Research and Systems Medicine, Department of Functional Sciences, “Victor Babeș” University of Medicine and Pharmacy Timișoara, 300041 Timișoara, Romania

**Keywords:** artificial intelligence, medical oncology, health literacy, diffusion of innovation, cohort studies

## Abstract

We examined whether baseline artificial intelligence (AI) economic literacy was associated with sustained use of oncology AI tools. This prospective cohort included 96 oncology and critical care professionals at two centres in Timișoara, Romania, followed for 12 months after institutional roll-out. Participants were classified by AI Economic Literacy Index (AIELI) scores as high (≥14) or low (<14). Sustained use required log-verified activity in at least two of the final three months. Analyses included regression, inverse probability of treatment weighting and exploratory indirect-association analysis through return-on-investment (ROI) confidence. Among 91 completers, sustained use occurred in 29/53 high-literacy participants (54.7%) and 7/38 low-literacy participants (18.4%; risk ratio 2.97, 95% confidence interval 1.46–6.06). The weighted risk ratio was 2.24 (1.15–4.34), and the profession-stratified risk ratio was 2.80 (1.36–5.76). Median time to first use was 5.8 months in the high-literacy group and was not reached in the low-literacy group. Nine participants (eight high-literacy, one low-literacy) had recorded use before the baseline assessment; in a delayed-entry analysis restricted to the 87 participants unused at assessment, the crude hazard ratio for subsequent first use was 2.98 (1.62–5.48) and the crude risk ratio for sustained use after excluding prior users was 3.01 (1.36–6.64); the corresponding covariate-adjusted estimates were less precise (hazard ratio 1.81, 0.89–3.69; risk ratio 2.19, 0.99–4.86) and their confidence intervals included the null. Roll-out-based time-to-first-use comparisons are descriptive because they include events preceding measurement. Sustained use increased across literacy bands. An indirect association through ROI confidence was observed but was not interpretable as causal mediation. Higher baseline economic literacy was associated with earlier and more sustained recorded AI-tool use, although opportunity for use differed by profession, and the adjusted timing-corrected estimates leave the independent contribution of literacy uncertain. Larger multicentre studies should evaluate whether literacy training improves adoption and whether adoption translates into clinical or economic benefit.

## Simple Summary

Artificial intelligence tools can support cancer care, but making them available does not ensure that healthcare professionals will use them regularly. We followed oncology staff after new tools were introduced at two centres in Timișoara, Romania, and compared their understanding of the costs and potential financial value of artificial intelligence with recorded tool use. Professionals with greater economic understanding used the tools earlier and more consistently, although the tools were more relevant to some professional roles than to others. Confidence in the value of the investment may help explain this association, although the study cannot establish cause and effect. These findings suggest that implementation programmes could test practical education about costs, expected benefits and reimbursement alongside technical training. Future studies should also assess whether regular use improves patient care and makes good use of healthcare resources.

## 1. Introduction

Artificial intelligence (AI) has moved from proof-of-concept studies to clinically deployable products across the oncology care pathway, including image-based tumour detection, automated segmentation for radiotherapy planning, natural-language processing of clinical documentation and risk prediction for treatment toxicity [1]. Cancer imaging in particular has become a leading domain for algorithmic support, with applications in screening, characterisation, response assessment and prognostication that are now embedded in commercially available platforms [2]. Yet the translation of validated algorithms into everyday practice remains uneven, and the gap between what AI systems can do in retrospective evaluations and how often they are actually used by clinicians has been identified as one of the defining challenges of medical AI in the current decade [3].

Experience with earlier technological innovations in oncology shows that clinical validity alone does not determine how quickly new approaches enter practice. Immune checkpoint inhibitors diffused unusually rapidly: in a large US real-world cohort, more than 60% of eligible patients with melanoma, non-small-cell lung cancer or renal cell carcinoma received anti-PD-1 therapy within four months of regulatory approval [4]. Precision diagnostics have spread far less evenly; across Europe, the use of multi-biomarker next-generation sequencing ranged from 0% to more than 50% between countries, and access to precision medicines was higher where public national reimbursement processes were in place [5]. Cell-based therapies such as chimeric antigen receptor T cells have been introduced through specialised centres under outcomes-based payment agreements negotiated separately in each health system [6], and therapeutic cancer vaccines, novel drug-delivery platforms and other personalised, data-driven interventions are likely to raise similar questions of infrastructure, evidence and payment. A recurring lesson is that innovations delivered through an established payment pathway, such as reimbursed medicines, tend to be adopted faster than those that require new infrastructure or lack a dedicated payment mechanism. Most oncology AI tools belong to the second group: they rarely generate a separately billable service [7], and an analysis of US insurance claims found that only a small number of AI procedures had dedicated billing codes, with recorded use concentrated in a few of them and in areas served by academic hospitals [8].

Economic considerations increasingly shape whether health systems, hospitals and individual departments elect to acquire and maintain AI tools. Systematic reviews of economic impact studies have found that most evaluations rely on modelling rather than prospective measurement, that costs of integration, maintenance and clinician time are frequently omitted, and that only a minority of studies satisfy established health-economic reporting standards [9]. A subsequent review of full health-economic evaluations described the field as a race between fast-moving technology and slow-moving evidence, noting that cost-effectiveness conclusions were sensitive to assumptions about workflow substitution and diagnostic accuracy [10]. Model-based analyses in melanoma, dental caries and diabetic retinopathy have shown that AI decision support can be cost-effective under some, but not all, care pathways and payer perspectives, reinforcing the importance of context-specific economic appraisal [11].

Beyond cost-effectiveness, reimbursement and payment mechanisms determine whether AI expenditure can be recovered. Analyses of payment for AI in medicine have emphasised that fee-for-service systems rarely accommodate software that improves efficiency without generating a billable event, so that return on investment (ROI) depends on institutional rather than procedural revenue [7]. Proposed reimbursement frameworks argue that AI should be paid for in proportion to demonstrated value, safety and equity, which requires evidence that most vendors and providers do not yet possess [12]. In radiology, the first coverage decisions for autonomous AI have illustrated how uncertainty about who pays, and for what, delays adoption even when clinical performance is established [13]. These payer-level uncertainties are transmitted to frontline professionals as perceived financial barriers and doubts about ROI.

In Romania, a multicentre cross-sectional survey of 108 oncology professionals introduced the AI Economic Literacy Index (AIELI) and found that higher economic literacy, greater ROI confidence and prior AI training were independently associated with stated willingness to invest in oncology AI, whereas perceived financial barriers were negatively associated [14]. The authors explicitly cautioned that their outcome captured stated support rather than enacted procurement or use, and that whether literacy-oriented capability development would increase actual adoption remained untested. This caution is consistent with integrative reviews of AI acceptance among hospital professionals, which have found that most evidence is cross-sectional and attitude-based, with very few longitudinal studies that follow professionals through an actual implementation [15]. Romanian surveys of internal medicine physicians have likewise documented highly favourable attitudes towards AI alongside low self-rated technical knowledge, a combination that leaves open whether favourable attitudes translate into use [16].

The distinction between intention and behaviour matters for implementation planning. Frameworks for the non-adoption, abandonment and failure to scale health technologies emphasise that adoption is a dynamic process shaped by the technology, the adopter system, the organisation and the wider economic environment, and that early attitudes are only one of several interacting determinants [17]. Psychological research has repeatedly shown that intentions explain only a modest share of subsequent behaviour, with roughly half of those who intend to act failing to do so, and that the strength of the intention–behaviour link depends on perceived control, confidence and habit formation [18]. Applied to oncology AI, this implies that the associations documented in cross-sectional surveys may overstate, understate or misattribute the determinants of real-world use, and that prospective designs with objectively verified outcomes are needed.

The present study addressed this gap with a prospective cohort design anchored to a natural implementation event: the institutional roll-out of licenced AI tools at two oncology centres in Timișoara, Romania. Its novelty lies in (i) measuring the AIELI at baseline near institutional roll-out, (ii) defining adoption by log-verified use rather than self-report, (iii) following professionals for 12 months, and (iv) applying confounding-adjustment methods, including propensity-score weighting and dose–response modelling, together with an exploratory analysis of indirect association, to a small but well-characterised cohort. The primary objective was to determine whether high baseline AI economic literacy predicts sustained AI-tool use at 12 months. Secondary objectives were to characterise time to first use, trajectories of ROI confidence and perceived financial barriers, and whether ROI confidence may contribute to the association between literacy and adoption.

## 2. Materials and Methods

### 2.1. Study Design, Setting and Population

This was a prospective, two-centre, observational cohort study conducted in Timișoara, western Romania, between 13 January 2025 and 31 March 2026. The participating centres were a public university-affiliated tertiary hospital oncology department and a private ambulatory oncology centre, both serving the Timiș county catchment area. Institutional licences for three AI tools accessible through single sign-on were activated on 6 January 2025 at the public hospital and on 13 January 2025 at the private centre (centre-specific roll-out dates): an automated organ-at-risk contouring module for radiotherapy planning (software version 2.4 throughout follow-up), an AI-supported clinical documentation assistant generating structured treatment summaries and coding suggestions (version 5.1, updated to version 5.2 in September 2025 without change to the logged event types) and a chemotherapy prescription safety-check module that flags dose and interaction issues (version 1.8). Product and vendor names are withheld in accordance with the institutional licence agreements and are recorded, with their version histories, in the protocol amendment (version 1.1, Section 3; chronology in Appendix A, Table A4). Roll-out was defined as activation of institutional accounts for eligible staff. Although every participant held an institutional account, the tools supported tasks performed predominantly by particular professional groups, such as radiotherapy contouring by radiation oncologists and prescription verification by prescribers and pharmacists. Account access therefore did not imply comparable opportunity for meaningful use. Tool interactions were recorded in institutional audit logs with pseudonymised user identifiers, permitting objective observation of recorded adoption. The tool-specific events that qualified as use, the delimitation of sessions and the definition of an active month are specified in Section 2.3 and Appendix A, Table A3. The study was designed and reported following the Strengthening the Reporting of Observational Studies in Epidemiology (STROBE) recommendations for cohort studies.

All oncology professionals employed at either centre for at least 0.5 full-time equivalents at the time of roll-out were invited to participate during the enrolment period from 13 January to 14 March 2025. The target population comprised medical oncologists, radiation oncologists, oncology nurses, hospital pharmacists and departmental administrators or health-economics staff. These roles encompass clinical, pharmacy and organisational responsibilities relevant to oncology AI implementation. A sample of approximately 100 participants was considered feasible given the combined workforce of roughly 130 eligible staff. Assuming sustained adoption in 50% of the high-literacy group and 20% of the low-literacy group, with a 3:2 allocation ratio, a two-sided α of 0.05 and 96 participants, the study had approximately 87% power to detect the primary difference.

### 2.2. Eligibility Criteria and Group Allocation

Inclusion criteria were: (i) age 18 years or older; (ii) direct involvement in oncology care delivery, pharmacy services or departmental management at a participating centre; (iii) an active institutional account with access to at least one of the three AI tools; and (iv) written informed consent to link survey responses to pseudonymised usage logs. Exclusion criteria were: (i) planned departure or retirement within 12 months; (ii) rotational trainees present for fewer than six months; (iii) participation in the vendor’s pilot programme before institutional roll-out, because such individuals would have had non-zero exposure at baseline; and (iv) inability to complete the questionnaires in Romanian or English. Of 131 eligible professionals, 96 (73.3%) consented and completed the baseline assessment and constitute the analysed cohort.

Exposure groups were defined by the baseline AI Economic Literacy Index (AIELI), a 25-point index assessing knowledge of cost structures, reimbursement pathways, return-on-investment concepts and value-based purchasing as applied to oncology AI. The index is also described in a Romanian cross-sectional survey published after this cohort’s baseline assessment [14]. For the present analysis, scores of 14 or more points defined the high-literacy group and scores below 14 defined the low-literacy group. This threshold was used as an analytical grouping rather than a validated clinical cut-off. Group allocation was observational rather than randomised. Multivariable adjustment and propensity-score weighting addressed measured differences between groups. Analyses using an alternative cut-off of 15 points, four literacy bands and a continuous AIELI specification assessed the robustness of the association to the exposure definition.

### 2.3. Variables, Outcomes and Data Collection

Baseline data were collected by electronic questionnaire during enrolment after institutional roll-out (baseline questionnaire version 1.0, dated 20 December 2024, with its 0–25 AIELI scoring key, as summarised in Appendix A, Table A4; the questionnaire and scoring key were administered unchanged to all participants, and the electronic form recorded a completion timestamp for each participant) and comprised age, sex, profession, sector, years of professional experience, weekly patient contacts, prior formal AI training (any accredited course of at least four hours), self-rated digital literacy (1–10), the AIELI (0–25), ROI confidence (0–10 numerical rating of confidence that institutional AI investment would generate financial or efficiency returns within three years), perceived financial barriers (five items scored 1–5 and summed to 5–25, higher indicating greater barriers) and willingness to invest in oncology AI (1–5). ROI confidence was re-measured at 3, 6, 9 and 12 months; perceived barriers and the AIELI were re-measured at 6 and 12 months; willingness to invest was re-measured at 12 months. Questionnaires were administered by the study coordinator, who was blinded to usage logs.

The chronology of study documents and analytical specifications is summarised in Appendix A, Table A4. The study protocol (version 1.0, 15 December 2023) was approved by the ethics committee on 30 January 2024 (approval code 044) and was amended before enrolment (version 1.1, 8 January 2025) to record the licenced tools and their versions, the centre-specific roll-out dates, the audit-log extraction procedure and the tool-specific events counted as use. The protocol and the baseline questionnaire were fixed before data collection, the AIELI items and scoring key, the 12-month follow-up, the questionnaire schedule and the log-based primary outcome. The dichotomisation of the AIELI at 14 points, the alternative cut-off of 15 points, the literacy bands, the propensity-score model, the imputation procedures and the exploratory indirect-association analysis were specified in a statistical analysis plan dated 24 March 2026, after baseline data collection but before the final 12-month log extraction and linkage (3 April 2026); they are therefore analytical choices made after enrolment rather than contemporaneous specifications. The Mantel–Haenszel analyses and the analyses addressing the timing of the baseline assessment relative to first use were added post hoc during peer review.

The primary outcome was sustained AI-tool use at 12 months, defined as log-verified activity with at least one of the three tools in at least two of the final three months of follow-up (months 10–12, counted from the centre-specific roll-out date rather than from individual enrolment). Secondary outcomes were: any documented use during follow-up; time from roll-out to first documented use (in months, at quarter-month resolution), with censoring at 12 months or at the date of loss to follow-up; the number of AI-tool sessions per month averaged over the final quarter; and 12-month ROI confidence, perceived financial barriers, willingness to invest and AIELI scores. Usage data were extracted monthly from the institutional audit logs by an information-technology officer and merged with questionnaire data using the pseudonymised identifier. Operational definitions of logged use are given in Appendix A, Table A3. A qualifying event was a tool-specific action that generated an output for a patient record: generation of an automatic organ-at-risk contour set in the contouring module, generation of a structured treatment summary or coding suggestion in the documentation assistant, and user-initiated submission of a chemotherapy prescription for checking in the safety-check module. Single sign-on log-ins without a qualifying action, navigation and page views, access to help pages and viewing of previously generated outputs were present in the audit logs but were not counted as use. Training and test interactions took place in a separate training tenant with fictitious patient identifiers, and their events were written to a separate training log that was not part of the production audit-log extracts; no further manual exclusions were applied to the production records. Qualifying events by the same user in the same tool separated by less than 30 min were grouped into one session, and activity in a second tool was counted as a separate session. An active month was a 30-day period counted from the centre-specific roll-out date in which at least one session was recorded in any tool; sustained use required an active month in at least two of months 10–12; the final-quarter session count was the mean number of sessions per 30-day period in months 10–12; and first use was the timestamp of the first qualifying event in any tool. Participants who left the institution were censored at their last month of employment and were treated as having missing 12-month questionnaire outcomes.

### 2.4. Statistical Analysis

Continuous variables are summarised as mean ± standard deviation (SD) when approximately normally distributed (assessed with the Shapiro–Wilk test and inspection of Q–Q plots) or as median with interquartile range (IQR) otherwise, and categorical variables as counts and percentages. Between-group comparisons used Welch’s *t*-test or the Mann–Whitney U test for continuous variables and the chi-square test, or Fisher’s exact test when any expected cell count was below five, for categorical variables. Standardised mean differences (SMD) were calculated for all baseline covariates, with absolute values below 0.10 regarded as adequate balance. The primary outcome was compared as a risk ratio (RR) and risk difference (RD) with Wald 95% confidence intervals (CI); adjusted RRs were obtained from modified Poisson regression with robust (HC1) standard errors. Longitudinal questionnaire scores were analysed with linear mixed models including a random intercept for participant and fixed effects for group, time and their interaction; within-group changes were additionally tested with paired *t*-tests. Time to first use was described with Kaplan–Meier estimates and compared with the log-rank test, and hazard ratios (HR) were estimated from Cox proportional hazards models whose proportional hazards assumption was verified with scaled Schoenfeld residual tests. Multivariable logistic regression estimated adjusted odds ratios (OR) for sustained use, and model performance was evaluated with the area under the receiver operating characteristic curve (AUC) with bootstrap optimism correction (500 resamples), the Hosmer–Lemeshow test, Nagelkerke’s R^2^, calibration slope and intercept, the Brier score and decision-curve analysis of the net benefit of classifying professionals as likely sustained users, computed in the development data without optimism correction (apparent net benefit). Multicollinearity was screened with variance inflation factors. Spearman’s rank correlation coefficients (ρ) described monotonic associations among ordinal and skewed variables. Subgroup analyses of the primary outcome examined profession, sector, age, prior AI training and baseline ROI confidence, with heterogeneity assessed by likelihood-ratio tests for exposure-by-subgroup interaction terms.

Three advanced analyses were conducted to address confounding, functional form and a possible indirect pathway. First, a propensity score for high AIELI was estimated by logistic regression from age, sex, profession, sector, prior AI training, digital literacy, years of experience and weekly patient contacts; stabilised inverse probability of treatment weights (IPTW) targeting the average treatment effect were truncated at the 99th percentile, covariate balance was re-assessed with weighted SMDs, and weighted RRs and HRs were estimated with robust standard errors. A doubly robust estimate combined weighting with covariate adjustment, and the E-value quantified the minimum strength of unmeasured confounding required to explain away the weighted RR. Analysis populations and methods for the estimates in Section 3.8 are summarised in Appendix A, Table A2. Second, the dose–response relationship between baseline literacy and sustained use was examined across four AIELI bands (≤9, 10–13, 14–17 and ≥18 points), with Wilson 95% CIs for band-specific proportions and a logistic test for linear trend across the ordered bands; the adjusted Cox model additionally included the AIELI as a continuous variable (per 5 points). Third, an exploratory analysis used the counterfactual mediation framework to decompose the association between high AIELI and sustained use into an indirect component through 6-month ROI confidence and a remaining direct component, using a linear mediator model and a logistic outcome model adjusted for age, sector, profession, prior training and digital literacy, with Monte Carlo integration over the mediator distribution and 95% percentile CIs from 1000 bootstrap resamples; moderated mediation by sector was examined through stratified conditional effects and their difference.

The AIELI, baseline ROI confidence and covariates were measured together at baseline; tool use could begin before the 6-month mediator assessment; and the outcome was assessed in months 10–12. Because early use may be affected by literacy and may in turn influence both 6-month confidence and later sustained use, it is a potential exposure-induced mediator–outcome confounder. Under this structure, natural indirect effects are not identified even though the outcome is measured after the mediator; the indirect estimate is therefore interpreted as an exploratory indirect association rather than an identified causal pathway, and the proportion mediated is not interpreted as a causal quantity. Missing 12-month outcomes arising from loss to follow-up (5.2%) were handled by complete-case analysis for the primary estimate, with multiple imputation by chained Equations (20 imputations, Rubin’s rules) and a worst-case imputation as sensitivity analyses; sporadic item-level missingness in the 6-month mediator (3 participants) was handled by listwise deletion in the mediation model only. All tests were two-sided; statistical significance was set at *p* < 0.05 without adjustment for multiplicity in secondary analyses, which are interpreted as exploratory, and exact *p*-values are reported to three decimal places. Analyses were performed in Python 3.11 (Python Software Foundation, Wilmington, DE, USA) (statsmodels 0.15, lifelines 0.30, scikit-learn 1.8).

**Figure 1:**
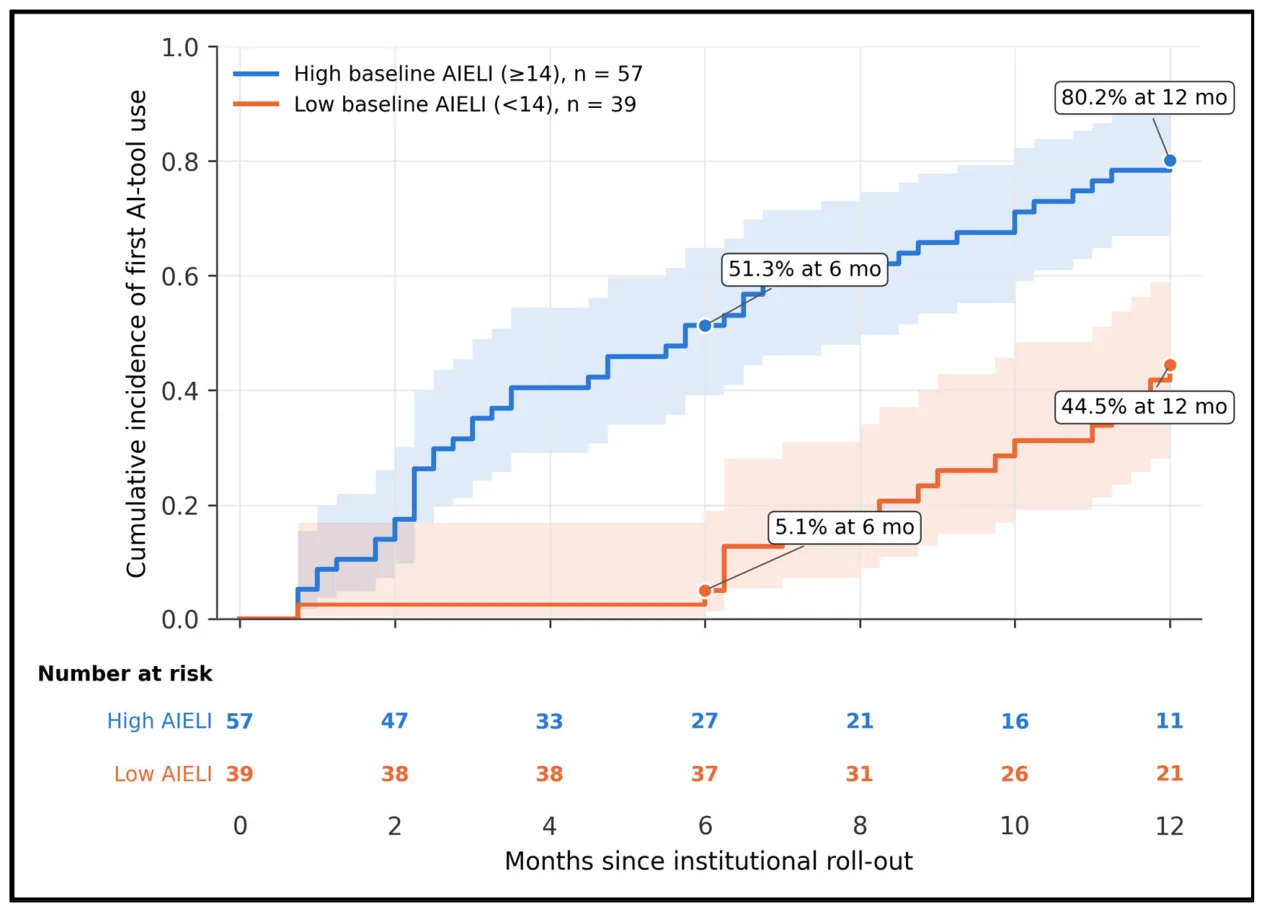
Kaplan–Meier cumulative incidence of first documented AI-tool use during 12 months of follow-up according to baseline AIELI group, with 95% confidence bands (shaded) and cumulative incidence at 6 and 12 months. Numbers at risk are shown below the plot. Time is measured from centre-specific roll-out. The curves are descriptive and include nine first-use events that preceded the baseline assessment (Appendix A, Table A5). AIELI, AI Economic Literacy Index.

Post hoc analyses added during peer review comprised Mantel–Haenszel RRs with Greenland–Robins variance, stratified by profession and restricted to physicians and nurses. These analyses provided a parsimonious comparison within broadly comparable professional task groups. Post hoc analyses also addressed the timing of the baseline assessment relative to first use. The questionnaire-completion timestamp of each participant was compared with the timestamp of the first qualifying event to identify recorded use before assessment. For subsequent initiation, a Cox model with delayed entry (left truncation) retained roll-out as the time scale but entered each participant into the risk set on the date of questionnaire completion and excluded participants with recorded use before assessment, so that only initiation after literacy measurement contributed events; a complementary model used questionnaire completion as the time origin with administrative censoring at the end of the 12-month roll-out-based follow-up. The primary sustained-use comparison was repeated after excluding participants with recorded use before assessment. The roll-out-based Kaplan–Meier and Cox analyses (Figure 1, Section 3.7) are retained as descriptive comparisons that include events preceding measurement. Secondary, subgroup and indirect-association analyses are interpreted as exploratory.

## 3. Results

### 3.1. Cohort Assembly and Baseline Characteristics

Of 131 eligible professionals, 96 were enrolled between 13 January and 14 March 2025 and followed for 12 months; 57 (59.4%) were classified as having high baseline AIELI (mean 16.6 ± 2.5) and 39 (40.6%) as low (mean 10.0 ± 2.6). Five participants (5.2%) left their institution during follow-up (four in the high-literacy group and one in the low-literacy group) and contributed usage data up to censoring; 91 (94.8%) completed the 12-month assessment. The baseline questionnaire was completed a median of 24 days (IQR 11–41; range 0–66) after the centre-specific roll-out date. Sixty-two participants (64.6%) had at least one documented AI-tool session during follow-up, and 36 of 91 completers (39.6%) met the definition of sustained use.

Baseline characteristics are summarised in Table 1. Mean age was 42.3 ± 9.9 years; 64.6% of participants were women and 41.7% were oncology nurses. High-literacy participants were younger than low-literacy participants (40.3 ± 9.4 vs. 45.2 ± 9.9 years; *p* = 0.019), had fewer years of professional experience (median 15.0 vs. 20.0; *p* = 0.036), and reported higher baseline ROI confidence (4.6 vs. 3.1; *p* < 0.001) and lower financial barriers (16.4 vs. 18.6; *p* = 0.001). Prior training and digital literacy had standardised mean differences of 0.35 and 0.39, respectively. Loss to follow-up was 7.0% versus 2.6% (*p* = 0.645).

Enrolment, follow-up completion and sustained use by profession and centre are shown in Appendix A, Table A1. All 96 participants had institutional access to at least one AI tool. Sustained use was observed in 17/34 physicians (50.0%), 13/38 oncology nurses (34.2%) and 6/19 pharmacists or administrators (31.6%), and in 19/49 (38.8%) and 17/42 (40.5%) completers at the public hospital and private centre, respectively.

### 3.2. Primary and Secondary Outcomes

Primary and secondary outcomes are presented in Table 2. Sustained use occurred in 29/53 high-literacy completers (54.7%) and 7/38 low-literacy completers (18.4%; RR 2.97, 95% CI 1.46–6.06; RD 36.3 percentage points, 95% CI 18.1–54.5; *p* < 0.001). Any documented use occurred in 78.9% versus 43.6% of enrolled participants. Median time to first use was 5.8 months in the high-literacy group and was not reached in the low-literacy group. Median monthly sessions in the final quarter were 5.0 versus 0.0 (*p* < 0.001). At 12 months, high-literacy participants had higher ROI confidence and willingness to invest and lower perceived financial barriers (all *p* < 0.001).

Figure 1 shows Kaplan–Meier estimates of the cumulative incidence of first documented use. At 6 months, cumulative incidence was 51.3% in the high-literacy group and 5.1% in the low-literacy group; at 12 months, the corresponding estimates were 80.2% and 44.5% (log-rank χ^2^ = 18.7; *p* < 0.001). The crude HR was 3.25 (95% CI 1.85–5.71). At 12 months, 11 high-literacy and 21 low-literacy participants remained at risk.

### 3.3. Longitudinal Change in Economic Attitudes

Longitudinal changes are presented in Table 3. ROI confidence increased by 1.4 points in the high-literacy group and 0.6 points in the low-literacy group; the 12-month group-by-time interaction was 0.9 points (95% CI 0.3–1.4; *p* = 0.003). The corresponding interaction was −0.9 points for perceived financial barriers (95% CI −1.8 to 0.1; *p* = 0.070) and 0.0 points for AIELI (95% CI −0.9 to 1.0; *p* = 0.967). AIELI increased by 2.3 points in each group.

**Figure 2:**
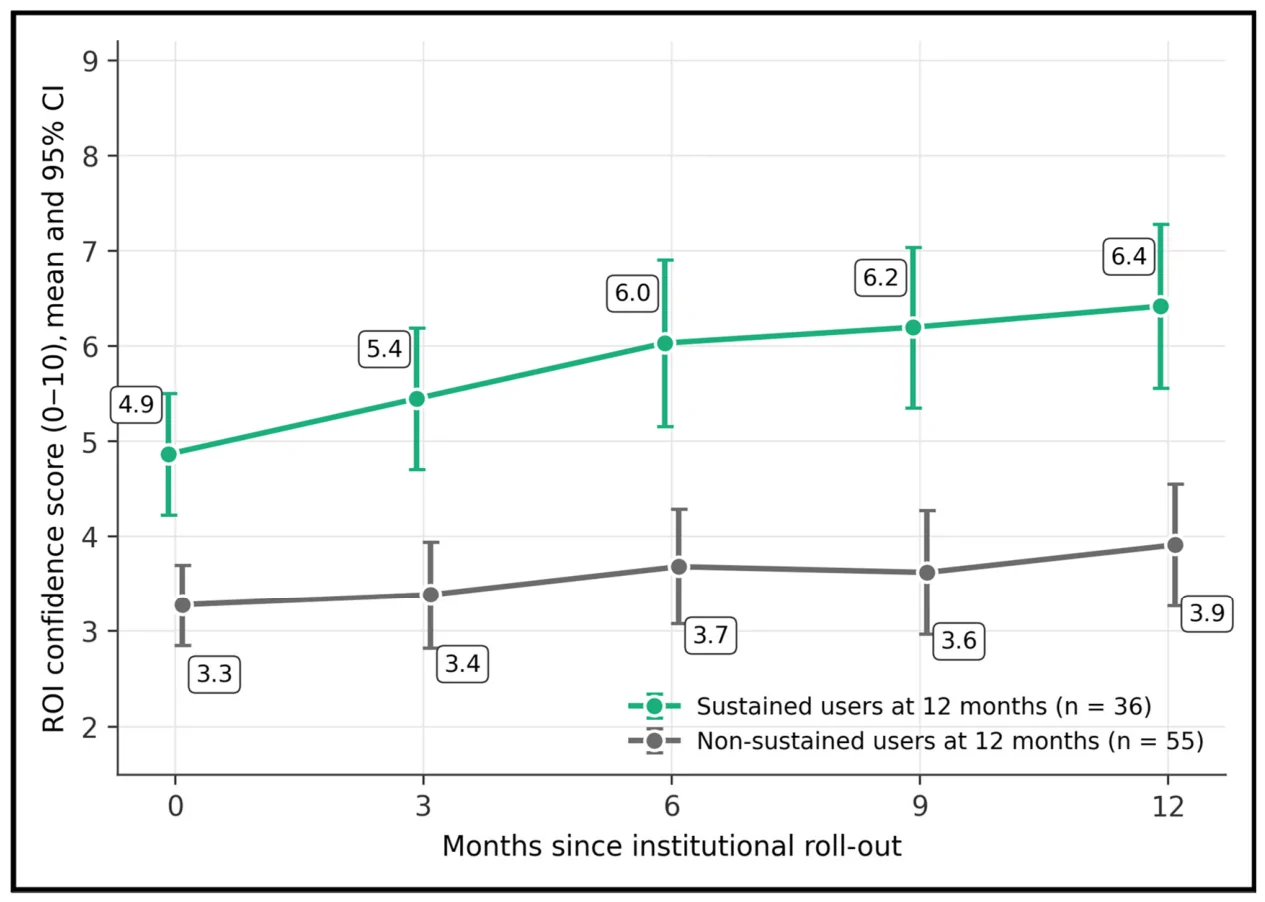
ROI-confidence trajectories (mean and 95% CI) at baseline, 3, 6, 9 and 12 months according to sustained AI-tool use at 12 months (completers, n = 91). ROI, return on investment; CI, confidence interval.

Figure 2 presents ROI-confidence trajectories according to sustained-use status at 12 months. Mean confidence increased from 4.9 to 6.4 points among sustained users and from 3.3 to 3.9 points among non-sustained users. The group-by-time interaction was 0.08 points per month (95% CI 0.02–0.14; *p* = 0.008). The between-group mean difference was 1.6 points at baseline and 2.5 points at 12 months.

### 3.4. Subgroup Analyses

Subgroup results are presented in Table 4. Risk ratios ranged from 1.77 to 3.95, with no statistically significant exposure-by-subgroup interactions (all *p* ≥ 0.428). Among participants with prior AI training, sustained use occurred in 72.0% of the high-literacy group and 25.0% of the low-literacy group. The RR was 3.68 (95% CI 1.23–10.98) in the public hospital and 2.44 (95% CI 0.95–6.24) in the private centre. Among participants with low baseline ROI confidence, the corresponding sustained-use proportions were 35.7% and 12.9%.

### 3.5. Predictors of Sustained Use

Logistic regression results are presented in Table 5. The univariable OR for sustained use was 3.82 per 5-point increase in baseline AIELI (95% CI 1.88–7.75). In the multivariable model, baseline ROI confidence was associated with sustained use (adjusted OR 1.54 per point, 95% CI 1.04–2.28; *p* = 0.032), whereas the adjusted AIELI estimate was 1.39 (95% CI 0.58–3.37; *p* = 0.463). Apparent and optimism-corrected AUCs were 0.837 and 0.786, respectively. The bootstrap calibration slope was 0.74, the intercept was −0.07, and the Brier score was 0.160.

**Figure 3:**
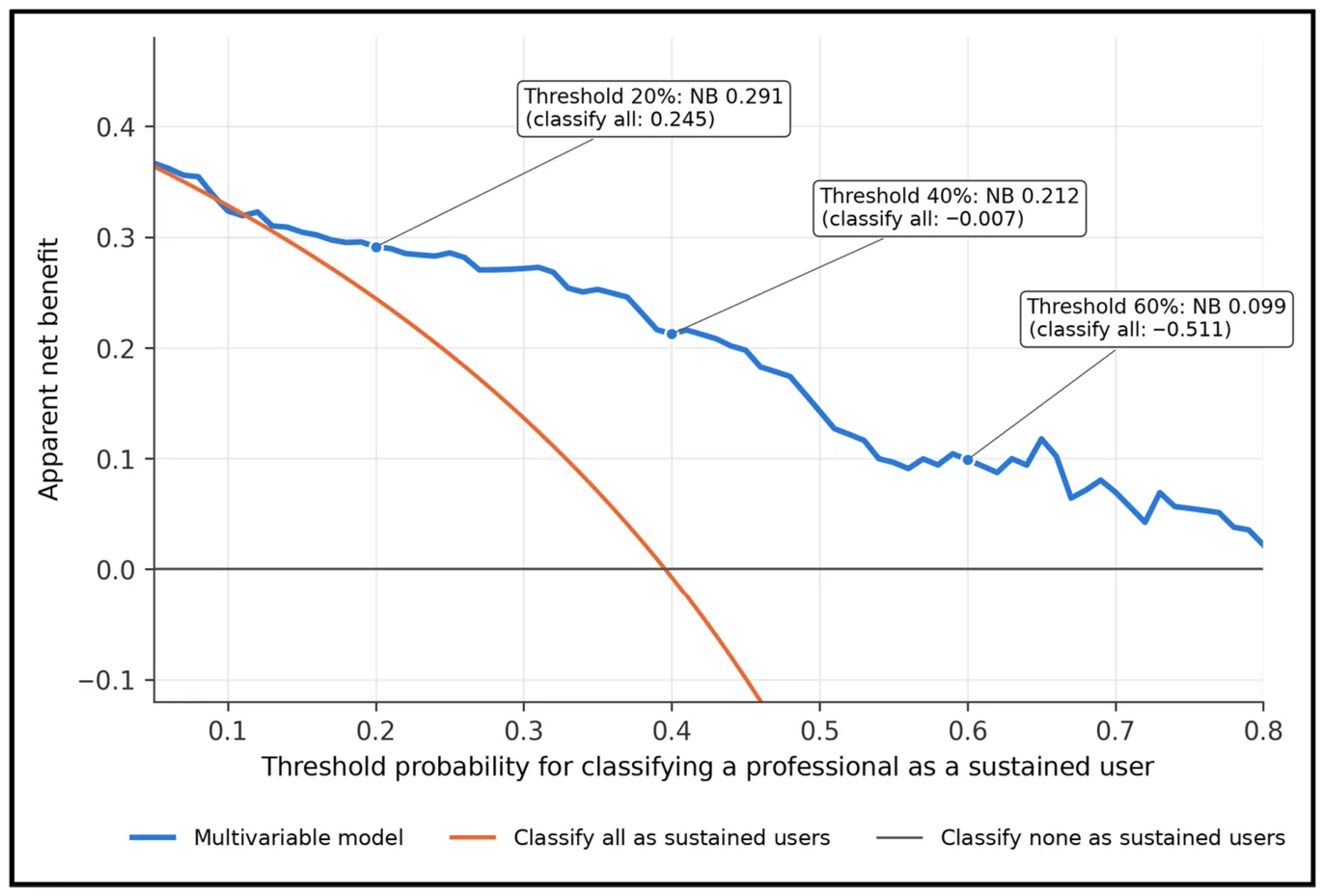
Decision-curve analysis of the multivariable model for sustained AI-tool use, comparing the net benefit of model-guided classification of sustained users with classifying all or no professionals as sustained users across threshold probabilities; labelled points give the net benefit at 20%, 40% and 60% thresholds. The horizontal axis gives the threshold probability for classifying a professional as a sustained user. Net benefits are apparent (in-sample) estimates without optimism correction; the figure does not identify who would benefit from implementation support. NB, net benefit.

Figure 3 shows the decision-curve analysis. At threshold probabilities of 20%, 40% and 60%, the apparent (in-sample) net benefits were 0.291, 0.212 and 0.099 for the model, compared with 0.245, −0.007 and −0.511 for the classify-all strategy. The classify-none strategy had a net benefit of zero. Model net benefit remained positive across the examined thresholds up to 80%. These values were not optimism-corrected; given the difference between apparent and optimism-corrected AUC (0.837 vs. 0.786) and the calibration slope of 0.74, they are likely to overstate net benefit in new data. The curve describes classification of sustained use and does not indicate who would benefit from implementation support.

### 3.6. Correlations Among Baseline Attitudes and Usage Intensity

Spearman correlations are presented in Table 6. Baseline AIELI correlated with ROI confidence (ρ = 0.50), willingness to invest (ρ = 0.43) and perceived financial barriers (ρ = −0.41; all *p* < 0.001). Final-quarter usage intensity correlated with baseline AIELI (ρ = 0.42; *p* < 0.001), baseline ROI confidence (ρ = 0.39; *p* < 0.001) and change in ROI confidence (ρ = 0.31; *p* = 0.003). The correlation between digital literacy and usage intensity was 0.15 (*p* = 0.148).

### 3.7. Time to First Documented Use

Cox regression results are presented in Table 7. Among 96 participants, 62 first-use events were recorded. The adjusted HR was 1.54 per 5-point increase in baseline AIELI (95% CI 1.02–2.31; *p* = 0.040); for high versus low AIELI, the adjusted HR was 1.93 (95% CI 0.98–3.77; *p* = 0.055). Prior training (adjusted HR 2.61; *p* = 0.002), physician status (adjusted HR 1.89; *p* = 0.030), and age (adjusted HR 0.68 per decade; *p* = 0.010) were also associated with time to first use. The adjusted model C-index was 0.747, and all scaled Schoenfeld residual test *p*-values were ≥0.12.

The timing of the baseline assessment relative to first use is summarised in Appendix A, Table A5. Nine of the 62 first-use events (14.5%) preceded questionnaire completion: 8/57 high-literacy participants (14.0%) and 1/39 low-literacy participants (2.6%) had recorded use before assessment, with a median of 9 days (range 2–31) before completing the questionnaire. Among the 87 participants unused at assessment (49 high-literacy and 38 low-literacy), 53 first-use events were recorded after assessment (37 and 16, respectively). In the delayed-entry Cox model, the crude HR for high versus low AIELI was 2.98 (95% CI 1.62–5.48; *p* < 0.001) and the adjusted HR was 1.81 (95% CI 0.89–3.69; *p* = 0.102); the adjusted HR for the continuous AIELI was 1.49 per 5 points (95% CI 0.97–2.28; *p* = 0.068). With questionnaire completion as the time origin, the crude HR was 2.91 (95% CI 1.59–5.33). All nine participants with prior use completed follow-up, and eight of them (seven high-literacy and one low-literacy) met the sustained-use definition. After their exclusion, sustained use occurred in 22/45 high-literacy completers (48.9%) and 6/37 low-literacy completers (16.2%; RR 3.01, 95% CI 1.36–6.64; *p* = 0.002; covariate-adjusted RR 2.19, 95% CI 0.99–4.86; *p* = 0.054).

**Figure 4:**
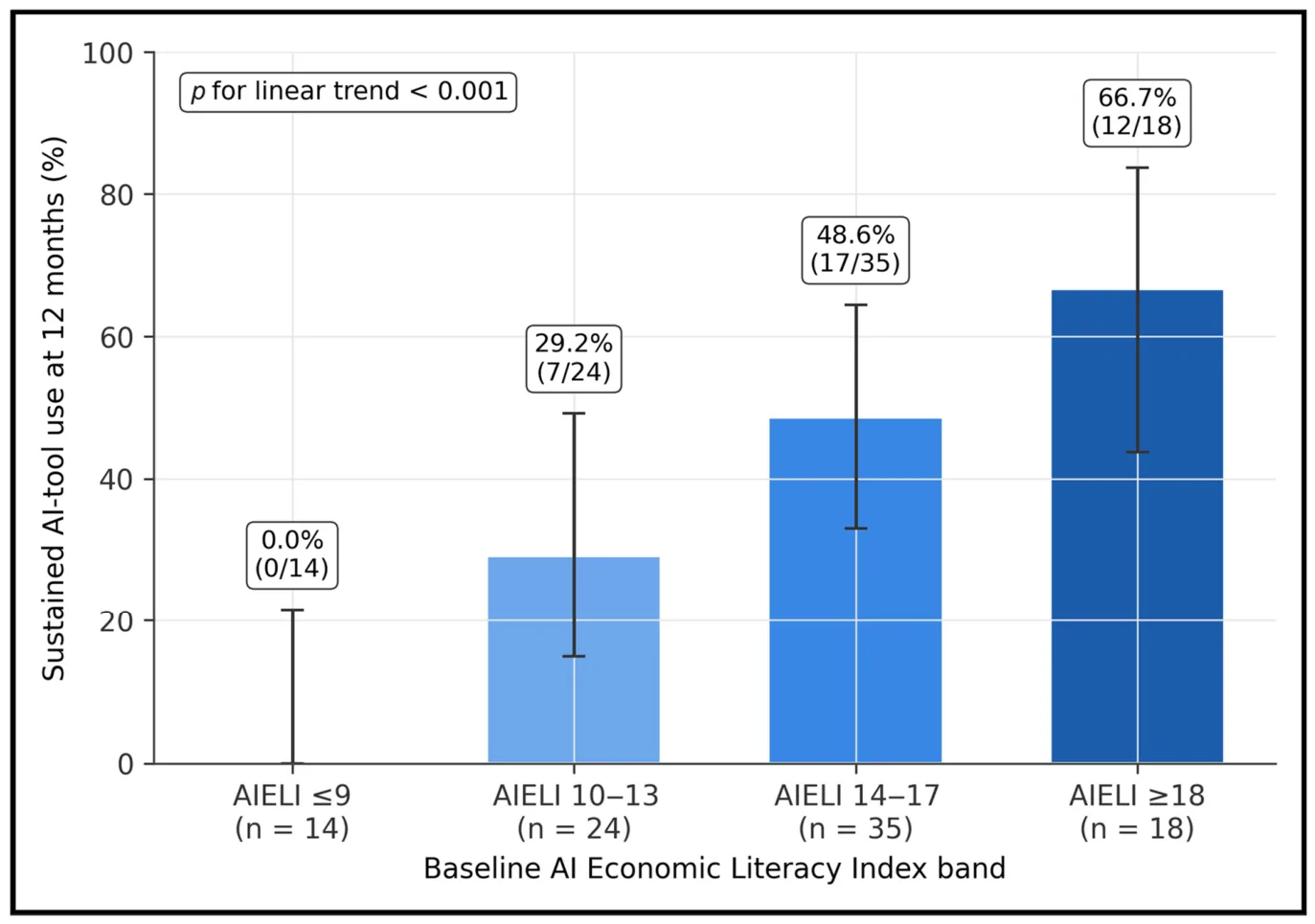
Proportion of participants with sustained AI-tool use at 12 months according to baseline AIELI band (completers, n = 91), with Wilson 95% confidence intervals and the *p*-value for linear trend across ordered bands. AIELI, AI Economic Literacy Index.

Figure 4 presents sustained use across baseline AIELI bands. Sustained use occurred in 0/14 participants scoring ≤9 points (0.0%, 95% CI 0.0–21.5%), 7/24 scoring 10–13 (29.2%, 95% CI 14.9–49.2%), 17/35 scoring 14–17 (48.6%, 95% CI 33.0–64.4%) and 12/18 scoring ≥18 (66.7%, 95% CI 43.7–83.7%). The OR per increase in one ordered band was 2.97 (95% CI 1.68–5.23; *p* for linear trend < 0.001).

### 3.8. Propensity-Score Weighting and Sensitivity Analyses

Weighting and sensitivity analyses are summarised in Table 8. The propensity-score model was fitted in all 96 enrolled participants, and the resulting weights were applied without re-estimation to the 91 completers for the sustained-use estimates and to all 96 participants for the time-to-first-use estimate (Appendix A, Table A6). Propensity scores ranged from 0.26 to 0.93 in the high-literacy group and from 0.08 to 0.87 in the low-literacy group; 87 participants (90.6%) lay within the region of common support (0.26–0.87), and the nine outside it (two high-literacy and seven low-literacy) were retained with stabilised weights between 0.44 and 0.65. Stabilised weights ranged from 0.44 to 2.58, and the weighted effective sample size was 84.6 of 96 enrolled participants (50.2 high-literacy and 31.9 low-literacy) and 80.3 of 91 completers (47.0 and 30.7). After weighting, the absolute SMD was ≤0.08 for all listed covariates except physician status (0.20). In the doubly robust model, profession entered the weighted outcome model as a three-level covariate, so that the residual physician imbalance was addressed by outcome-model adjustment; this adjustment balances recorded professional group but does not remove differences in task opportunity between professions. In a post hoc Mantel–Haenszel analysis stratified by profession, the RR was 2.80 (95% CI 1.36–5.76; *p* = 0.005); restricted to physicians and nurses (n = 72), it was 2.82 (95% CI 1.23–6.46; *p* = 0.015). The covariate-adjusted, IPTW-weighted and doubly robust RRs were 2.25 (95% CI 1.07–4.74), 2.24 (95% CI 1.15–4.34) and 2.07 (95% CI 1.04–4.12), respectively. Multiple imputation yielded an RR of 2.89 (95% CI 1.42–5.88), and worst-case imputation yielded an RR of 2.48 (95% CI 1.27–4.84). At the alternative AIELI cut-off of ≥15, the adjusted RR was 1.68 (95% CI 0.89–3.15). The literacy-band results are shown in Figure 4. The E-value was 3.90 for the IPTW point estimate and 1.57 for its lower confidence limit. Analysis populations and methods are summarised in Table A2.

### 3.9. Exploratory Indirect Association Through Return-on-Investment Confidence

Results of the exploratory indirect-association analysis are presented in Table 9. Among 88 participants with complete mediator data, the estimated total association on the risk-difference scale was 0.284 (95% CI 0.052–0.473), the indirect association through 6-month ROI confidence was 0.137 (95% CI 0.023–0.261; *p* = 0.020), and the direct association was 0.147 (95% CI −0.083 to 0.365; *p* = 0.202). The ratio of the indirect to the total estimate was 48.2%, but its 95% CI (4.3–189.6%) extended beyond the range of a proportion, and it is not interpreted as the share of the association transmitted through ROI confidence. The sector-specific indirect estimates were 0.182 for the public hospital and 0.058 for the private centre; the index of moderated mediation was −0.125 (95% CI −0.383 to 0.106; *p* = 0.336).

## 4. Discussion

### 4.1. Principal Findings and Comparison with Previous Literature

Higher baseline economic literacy was associated with sustained, log-verified AI-tool use after institutional roll-out. The crude association weakened after adjustment and weighting, indicating that differences in measured characteristics contributed to the unadjusted result. The weighted estimates remained compatible with an association, but the alternative dichotomous cut-off and the fully adjusted logistic model produced imprecise estimates. The findings therefore support economic literacy as a potential marker of implementation readiness, while leaving the magnitude and independence of its contribution uncertain. Because the three tools serve tasks of differing relevance to physicians, nurses, pharmacists and administrators, the association is best interpreted as one with recorded engagement under differing opportunities for use. It persisted when comparisons were confined to professional strata (Mantel–Haenszel RR 2.80) and to clinical staff, and when participants with recorded use before the baseline assessment were excluded (crude RR 3.01; covariate-adjusted RR 2.19, 95% CI 0.99–4.86), but the small strata precluded tool-specific comparisons within groups with identical access. This interpretation is consistent with technology-acceptance theory, which links perceived usefulness and ease of use to acceptance [19] and places performance expectations and facilitating conditions within a broader account of use behaviour [20].

The distinction between recorded use and favourable attitudes is central to interpreting this cohort. Reviews of healthcare professionals’ understanding of AI describe gaps in knowledge and limited evidence on experience with deployed systems [21]. Staff surveys identify uncertainty alongside interest [22], and stakeholder research emphasises practical value and the conditions under which AI is introduced [23]. The present log-based outcome addresses a different stage of implementation from the willingness-to-invest outcome in the Romanian AIELI survey [14]. Because the cohorts, outcomes and study designs differ, the cross-sectional association provides context rather than a directly comparable estimate or evidence that willingness necessarily leads to use.

Evidence from medical students also requires careful translation to established oncology teams. Sit and colleagues found that prior AI teaching was associated with greater confidence in working with AI, but their cross-sectional survey did not test sustained clinical use or an economic-literacy intervention [24]. The scoping review by Charow and colleagues described heterogeneous education programmes and evaluation approaches, supporting the need to assess behavioural and organisational outcomes alongside knowledge gains [25]. The multicentre student survey by Pinto dos Santos and colleagues similarly concerned attitudes and educational exposure rather than prospective adoption [26]. These studies justify testing education as an implementation component, but they do not establish that raising AIELI scores will reproduce the association observed here.

### 4.2. Economic Confidence and Organisational Context

Economic knowledge could help professionals distinguish acquisition costs from recurring operating costs, interpret reimbursement uncertainty and assess how workflow changes affect the value of a tool. These are plausible explanations for the association, rather than mechanisms directly demonstrated by this study. Interviews with Swedish healthcare leaders identified implementation challenges involving external conditions, organisational capacity and changes in professional practice [27]. Structured model information, such as the fact labels proposed by Sendak and colleagues, offers a complementary way to communicate intended use and limitations [28]. Accessible information and economic education may therefore address different but related sources of uncertainty. These explanations also presuppose that frontline staff can obtain relevant cost and return information, which is often not the case (Section 4.4).

The indirect-association findings are compatible with ROI confidence contributing to the literacy–adoption relationship, but they should not be interpreted as showing that a fixed share of adoption was caused by confidence. The confidence interval for the ratio of the indirect to the total estimate extended beyond the usual percentage range, reflecting instability when the component effects are imprecise. Moreover, the mediator was the 6-month confidence score, rather than a separately identified change score. Baseline confidence already differed by literacy group, and early tool use could itself increase confidence. By the 6-month assessment, approximately half of the high-literacy group had already initiated use (Figure 1), so early use may have influenced both 6-month confidence and later sustained use. Under the assumed structure (Section 2.4), early use is therefore an exposure-induced mediator–outcome confounder, and measuring the outcome after the mediator does not make the indirect effect identifiable. Adjustment for early use, a variable potentially affected by economic literacy, would not by itself recover a natural indirect effect. The analysis cannot fully separate pre-existing optimism, learning through experience and the subsequent consolidation of use. We therefore report an exploratory indirect association rather than a proportion of the effect mediated by ROI confidence.

Sector-specific estimates also require restraint. The indirect estimate was larger in the public hospital, but the interaction analysis did not establish a difference between sectors. With only one centre representing each sector, ownership is inseparable from centre-specific leadership, workflow, staffing and implementation arrangements. Qualitative research in diagnostic clinics highlights organisational readiness and implementation conditions [29]. In Dutch radiology departments, Strohm and colleagues identified local champions and hospital innovation strategies as facilitators, alongside barriers related to uncertain clinical value and unstructured implementation [30]. Such contextual factors could contribute to the associations observed in this cohort and should be measured explicitly in future studies.

### 4.3. Implications for Oncology Implementation

A practical implication is to evaluate economic education alongside technical onboarding, with examples drawn from the actual tools used by each professional group. Relevant content could include licensing and maintenance costs, time required to review outputs, the distinction between efficiency gains and recoverable revenue, and uncertainty in expected returns. Programmes could also give frontline professionals access to institution-specific information on licence costs, staff time and realised benefits, which clinicians often lack [31] and which hospital-level return-on-investment models show to depend strongly on local assumptions [32]. Implementation in resource-constrained settings must also consider affordability and institutional capacity [33], while governance frameworks emphasise responsibility and oversight [34]. These considerations suggest that education should support informed appraisal of a tool’s value, rather than encourage higher usage regardless of clinical need.

The literacy-band gradient suggests that a continuous measure may retain more information than a single cut-off. However, sparse data in the lowest band and overlapping confidence intervals limit claims about the functional form of the relationship. A low score could help identify topics for additional training, but it should not be used to restrict access to useful tools or to judge staff performance. Stepwise implementation approaches emphasise ongoing evaluation [35], and transparent assessment of effectiveness remains necessary throughout deployment [36]. In this cohort, the outcome recorded frequency and persistence of use; it did not establish appropriateness, better treatment decisions, reduced toxicity, improved documentation quality or realised financial returns.

The decision-curve analysis concerns classification of likely sustained users, not the causal benefit of giving a professional additional support. A person with a high predicted probability of use may adopt without intensive onboarding, whereas a person with a low probability may benefit substantially from it. Consequently, the curve cannot determine who should receive scarce training resources or quantify the number of additional adopters that an intervention would produce. Its interpretation also depends on calibration, the chosen outcome prevalence and the practical meaning of each threshold. External validation and a defined intervention are needed before this model could inform operational decisions. In addition, the net benefits shown in Figure 3 are apparent estimates from the development data; with an optimism-corrected AUC of 0.786 and a calibration slope of 0.74, internally validated net benefit would be expected to be lower.

### 4.4. Strengths and Limitations

The prospective follow-up, repeated confidence assessments, and linkage to institutional usage logs are strengths. Nevertheless, the small cohort and limited number of sustained-use events constrain precision, particularly for subgroup, mediation and multivariable analyses. The difference between apparent and optimism-corrected discrimination and the calibration slope below unity indicate model optimism. A non-significant Hosmer–Lemeshow test does not establish satisfactory calibration in a small sample. The prediction model therefore requires validation in independent institutions before its performance can be generalised.

Exposure was observational, and higher-literacy professionals differed in age, occupation, experience and prior training. Weighting reduced most measured imbalances but left residual imbalance in physician status, which profession-stratified analyses addressed only partly. Unmeasured workload, leadership, access opportunities and team influences may also affect both literacy and use. The E-value describes sensitivity to unmeasured confounding under its assumptions; it does not demonstrate the absence of confounding. Participants were drawn from two centres in one city, and non-participating staff may have differed in motivation or familiarity with AI, limiting transportability.

The AIELI cut-off requires further validation; findings from the alternative cut-off, continuous score and literacy bands should therefore be considered alongside the primary dichotomous comparison. Recruitment followed roll-out, and nine participants (eight in the high-literacy group) had recorded use before completing the baseline questionnaire, so early tool exposure may have influenced their baseline responses. The analyses restricted to participants unused at assessment, with entry into the risk set at questionnaire completion, gave associations of similar direction and magnitude, but the adjusted delayed-entry hazard ratio was imprecise, and the roll-out-based Kaplan–Meier and Cox estimates include these early events and should be read as descriptive comparisons rather than as prospective estimates for every participant. The three tools serve different tasks, and a combined any-tool endpoint may conceal differences in opportunity and clinical relevance across professions. Profession-stratified analyses address this only partly, because profession is an imperfect proxy for task-specific opportunity. Usage logs establish recorded activity but not the quality of the interaction or whether the output was accepted. Although qualifying events were restricted to output-generating actions (Table A3), the logs do not indicate whether an output was clinically relevant or acted upon. Self-reported confidence and perceived barriers are also vulnerable to response and expectation effects. Neither clinical outcomes nor actual costs and returns were measured, precluding conclusions about cost-effectiveness.

The association may also depend on the payment system in which the tools were introduced. Adoption of earlier oncology innovations has differed according to whether a reimbursement pathway existed [5,6], and AI tools seldom generate a separately billable service [7,8]. The two centres differed in ownership and, potentially, in payer mix, and the study did not record how the licences were financed or what financial information participants received about the tools. In many health systems, frontline oncology professionals have limited access to institution-specific data on costs, savings or return on investment [31], even though such returns depend on local assumptions about workflow and downstream activity [32], and they are nonetheless expected to adopt new tools quickly. This disconnect means that self-rated ROI confidence may reflect general expectations rather than informed appraisal, and the findings may not transfer to systems with dedicated reimbursement for AI or with different arrangements for sharing financial outcome data with clinicians.

Missing outcomes were uncommon, but complete-case analysis can still be biased if departure or non-response is related to adoption. Imputation and worst-case analyses explore selected assumptions without resolving all missing-data mechanisms. Secondary analyses were numerous and unadjusted for multiplicity, so isolated findings should be treated as exploratory. Mediation additionally requires assumptions about exposure–mediator and mediator–outcome confounding (including confounding induced by early use), temporal order and model specification that cannot be verified from the observed associations.

### 4.5. Future Research

The next step is a multicentre prospective study with baseline measurement before tool access, a published analysis plan, tool-specific usage definitions and explicit measurement of organisational support and of the financial information and payment arrangements available to staff, ideally across health systems with different payment models. Analyses should retain continuous literacy information, report calibration as well as discrimination, and account for clustering by centre and professional team. Repeated measurements of confidence before and after initial use would help clarify whether confidence precedes adoption, follows experience or participates in a feedback process.

A cluster-randomised or stepped-wedge evaluation could compare standard technical onboarding with onboarding supplemented by economic education. Outcomes should include appropriate use, time spent checking AI outputs, staff workload, patient safety, costs and realised benefits, alongside sustained adoption. Such a study could determine whether improving literacy changes behaviour and whether those changes deliver value for oncology services. Qualitative interviews with persistent users, late adopters and non-users would help explain how financial understanding interacts with tool performance and local working conditions.

## 5. Conclusions

Higher baseline AI economic literacy was associated with earlier and more sustained recorded use of oncology AI tools, although opportunities for use differed between professional groups. The association with initiation remained positive in direction when the risk set was restricted to professionals who had not used the tools before literacy was measured (crude hazard ratio 2.98), but the covariate-adjusted estimate was less precise and its confidence interval included the null (hazard ratio 1.81, 95% CI 0.89–3.69), so these analyses distinguish the observed association from evidence of an independent contribution of literacy; the roll-out-based time-to-first-use comparison is descriptive because it includes a small number of events that preceded measurement. An exploratory indirect association through ROI confidence was observed, but the observational design does not establish a causal mechanism. Economic literacy education merits evaluation alongside technical and organisational implementation support. Multicentre validation and intervention studies are needed to determine whether improving literacy promotes appropriate tool use and benefits cancer care.

## Figures and Tables

**Table 1:** Baseline characteristics of the cohort according to baseline AI Economic Literacy Index (AIELI) group.

Characteristic	All (n = 96)	High AIELI (n = 57)	Low AIELI (n = 39)	SMD	*p*-Value
Age, years	42.3 ± 9.9	40.3 ± 9.4	45.2 ± 9.9	−0.50	0.019
Female sex	62 (64.6)	40 (70.2)	22 (56.4)	0.29	0.166
**Profession, n (%)**					
Physician (medical/radiation oncologist)	35 (36.5)	26 (45.6)	9 (23.1)	0.49	0.074
Oncology nurse	40 (41.7)	21 (36.8)	19 (48.7)	−0.24	
Pharmacist/administrator	21 (21.9)	10 (17.5)	11 (28.2)	−0.26	
Private-sector centre	44 (45.8)	26 (45.6)	18 (46.2)	−0.01	0.958
Professional experience, years	15.5 (9.0–22.2)	15.0 (6.0–22.0)	20.0 (11.0–24.5)	−0.50	0.036
Prior formal AI training	39 (40.6)	27 (47.4)	12 (30.8)	0.35	0.104
Self-rated digital literacy (1–10)	6.8 ± 1.5	7.1 ± 1.5	6.5 ± 1.5	0.39	0.065
Weekly patient contacts	43.0 (30.8–53.2)	43.0 (28.0–54.0)	44.0 (34.5–50.5)	0.03	0.997
Baseline AIELI (0–25)	13.9 ± 4.1	16.6 ± 2.5	10.0 ± 2.6	2.59	<0.001
Baseline ROI confidence (0–10)	4.0 ± 1.8	4.6 ± 1.7	3.1 ± 1.7	0.88	<0.001
Baseline perceived financial barriers (5–25)	17.3 ± 3.3	16.4 ± 3.2	18.6 ± 2.9	−0.70	0.001
Baseline willingness to invest (1–5)	2.8 ± 1.0	3.1 ± 1.0	2.3 ± 0.9	0.86	<0.001
Lost to follow-up	5 (5.2)	4 (7.0)	1 (2.6)	0.21	0.645

Values are mean ± SD, median (IQR) or n (%). *p*-values from Welch’s t-test (age, digital literacy, AIELI, ROI confidence, barriers, willingness), the Mann–Whitney U test (experience, patient contacts), the chi-square test (sex, profession overall, sector, prior training) or Fisher’s exact test (loss to follow-up). SMD > 0 indicates higher values in the high-AIELI group. Abbreviations: AI, artificial intelligence; AIELI, AI Economic Literacy Index; IQR, interquartile range; ROI, return on investment; SD, standard deviation; SMD, standardised mean difference. n, number of participants.

**Table 2:** Primary and secondary outcomes at 12 months by baseline AIELI group.

Outcome	High AIELI (n = 53) ^a^	Low AIELI (n = 38) ^a^	Effect Estimate (95% CI)	*p*-Value
Sustained AI-tool use at 12 months (primary)	29 (54.7)	7 (18.4)	RR 2.97 (1.46–6.06); RD 36.3% (18.1 to 54.5)	<0.001
Any documented AI-tool use during follow-up	45 (78.9)	17 (43.6)	RR 1.81 (1.24–2.65)	<0.001
Median time to first documented use, months (95% CI)	5.8 (3.2–8.5)	NR (11.0–NR)	Log-rank test	<0.001
AI-tool sessions per month, final quarter	5.0 (0.0–8.0)	0.0 (0.0–1.0)	Mann–Whitney U	<0.001
12-month ROI confidence (0–10)	5.8 ± 2.6	3.6 ± 2.3	MD 2.3 (1.2 to 3.3); d = 0.92	<0.001
12-month perceived financial barriers (5–25)	14.8 ± 3.9	17.8 ± 4.2	MD −3.0 (−4.8 to −1.3); d = −0.75	<0.001
12-month willingness to invest (1–5)	3.5 ± 1.3	2.4 ± 1.2	MD 1.1 (0.6 to 1.6); d = 0.90	<0.001
12-month AIELI (0–25)	18.9 ± 3.6	12.2 ± 3.9	MD 6.7 (5.1 to 8.3); d = 1.79	<0.001

^a^ Completers with 12-month data; time-to-event and any-use outcomes include all 96 enrolled participants (57 high, 39 low). Values are n (%), median (IQR) or mean ± SD. RR and RD with Wald 95% CI and chi-square *p*-values; mean differences with Welch 95% CI and *p*-values; Cohen’s d for standardised differences. Abbreviations: AI, artificial intelligence; AIELI, AI Economic Literacy Index; CI, confidence interval; d, Cohen’s d; IQR, interquartile range; MD, mean difference; NR, not reached; RD, risk difference; ROI, return on investment; RR, risk ratio; SD, standard deviation. n, number of participants.

**Table 3:** Trajectories of ROI confidence, perceived financial barriers and AIELI over 12 months by exposure group (completers, n = 91).

Score	Group	Baseline	6 Months	12 Months	Within-Group Change 0–12 Months (95% CI)	*p* ^a^	Between-Group Difference in Change (95% CI)	*p* ^b^
ROI confidence (0–10)	High	4.5 ± 1.7	5.4 ± 2.5	5.8 ± 2.6	1.4 (0.9 to 1.8)	<0.001	0.9 (0.3 to 1.4)	0.003
	Low	3.1 ± 1.8	3.4 ± 2.2	3.7 ± 2.3	0.6 (0.1 to 1.1)	0.031		
Perceived financial barriers (5–25)	High	16.4 ± 3.1	15.2 ± 3.6	14.8 ± 3.9	−1.6 (−2.4 to −0.8)	<0.001	−0.9 (−1.8 to 0.1)	0.070
	Low	18.6 ± 3.0	18.1 ± 3.9	17.8 ± 4.2	−0.8 (−1.6 to 0.1)	0.079		
AIELI (0–25)	High	16.7 ± 2.5	18.3 ± 3.4	18.9 ± 3.6	2.3 (1.5 to 3.0)	<0.001	0.0 (−0.9 to 1.0)	0.967
	Low	9.9 ± 2.7	11.8 ± 3.5	12.2 ± 3.9	2.3 (1.4 to 3.2)	<0.001		

Values are mean ± SD. ^a^ Paired *t*-test of the 12-month change within each group. ^b^ Group-by-time interaction from a linear mixed model with a random intercept for participant (high vs. low AIELI at 12 months relative to baseline). Positive between-group differences indicate larger increases in the high-AIELI group. Abbreviations: AI, artificial intelligence; AIELI, AI Economic Literacy Index; CI, confidence interval; ROI, return on investment; SD, standard deviation. High and Low denote high and low baseline AIELI, respectively.

**Table 4:** Sustained AI-tool use at 12 months by baseline AIELI group within subgroups (completers, n = 91).

Subgroup	Stratum	High AIELI, n/N (%)	Low AIELI, n/N (%)	RR (95% CI)	*p*-Value	*p* for Interaction
Profession	Physician	15/26 (57.7)	2/8 (25.0)	2.31 (0.66–8.01)	0.225	0.949
	Oncology nurse	10/19 (52.6)	3/19 (15.8)	3.33 (1.08–10.24)	0.038	
	Pharmacist/administrator	4/8 (50.0)	2/11 (18.2)	2.75 (0.66–11.52)	0.319	
Sector	Public hospital	16/29 (55.2)	3/20 (15.0)	3.68 (1.23–10.98)	0.007	0.604
	Private centre	13/24 (54.2)	4/18 (22.2)	2.44 (0.95–6.24)	0.057	
Age	<40 years	17/28 (60.7)	2/13 (15.4)	3.95 (1.07–14.61)	0.009	0.428
	≥40 years	12/25 (48.0)	5/25 (20.0)	2.40 (0.99–5.81)	0.037	
Prior AI training	No	11/28 (39.3)	4/26 (15.4)	2.55 (0.93–7.03)	0.070	0.456
	Yes	18/25 (72.0)	3/12 (25.0)	2.88 (1.05–7.91)	0.012	
Baseline ROI confidence	≤4 (low)	10/28 (35.7)	4/31 (12.9)	2.77 (0.98–7.84)	0.065	0.915
	>4 (high)	19/25 (76.0)	3/7 (42.9)	1.77 (0.73–4.29)	0.165	

RR with Wald 95% CI; *p*-values from the chi-square test or Fisher’s exact test when any expected count was below five; *p* for interaction from likelihood-ratio tests comparing logistic models with and without exposure-by-subgroup terms. Abbreviations: AI, artificial intelligence; AIELI, AI Economic Literacy Index; CI, confidence interval; ROI, return on investment; RR, risk ratio. n, number with sustained use; N, number of participants in the stratum.

**Table 5:** Univariable and multivariable logistic regression models for sustained AI-tool use at 12 months (n = 91; 36 events).

Predictor	Univariable OR (95% CI)	*p*-Value	Adjusted OR (95% CI) ^a^	*p*-Value
Baseline AIELI, per 5 points	3.82 (1.88–7.75)	<0.001	1.39 (0.58–3.37)	0.463
Baseline ROI confidence, per point	1.76 (1.30–2.38)	<0.001	1.54 (1.04–2.28)	0.032
Baseline perceived barriers, per point	0.81 (0.70–0.94)	0.007	0.85 (0.70–1.04)	0.110
Prior formal AI training (yes vs. no)	3.41 (1.41–8.24)	0.006	2.47 (0.78–7.79)	0.124
Private-sector centre (vs. public)	1.07 (0.46–2.49)	0.869	0.50 (0.16–1.58)	0.238
Physician (vs. non-physician)	2.00 (0.84–4.77)	0.118	2.65 (0.82–8.56)	0.102
Age, per 10 years	0.64 (0.41–1.01)	0.055	0.65 (0.37–1.15)	0.141
Digital literacy, per point	1.38 (1.03–1.85)	0.033	—	—
Female sex	0.86 (0.36–2.08)	0.739	—	—

^a^ Multivariable model with the seven listed covariates entered simultaneously: likelihood-ratio χ^2^ = 33.1 (7 df), *p* < 0.001; apparent AUC 0.837, optimism-corrected AUC 0.786 (500 bootstrap resamples); Hosmer–Lemeshow χ^2^ = 7.5 (8 df), *p* = 0.485; Nagelkerke R^2^ = 0.413; bootstrap calibration slope 0.74 and intercept −0.07; Brier score 0.160; maximum VIF 1.89. Digital literacy and sex were not included in the multivariable model because of collinearity with AIELI and prior training and the absence of univariable association, respectively. Abbreviations: AI, artificial intelligence; AIELI, AI Economic Literacy Index; AUC, area under the receiver operating characteristic curve; CI, confidence interval; df, degrees of freedom; OR, odds ratio; ROI, return on investment; VIF, variance inflation factor. n, number of participants.

**Table 6:** Spearman rank correlation matrix among baseline attitudes, participant characteristics and 12-month usage intensity (completers, n = 91).

Variable	1	2	3	4	5	6	7	8
1. Baseline AIELI	—							
2. Baseline ROI confidence	0.50 ***	—						
3. Baseline barriers	−0.41 ***	−0.31 **	—					
4. Baseline willingness	0.43 ***	0.42 ***	−0.37 ***	—				
5. Digital literacy	0.25 *	0.27 *	−0.05	0.28 **	—			
6. Age	−0.29 **	−0.05	0.12	−0.12	−0.31 **	—		
7. Sessions/month (final quarter)	0.42 ***	0.39 ***	−0.30 **	0.23 *	0.15	−0.24 *	—	
8. Δ ROI confidence (0–12 mo)	0.25 *	0.14	−0.12	0.26 *	−0.15	0.13	0.31 **	—

Spearman ρ; * *p* < 0.05, ** *p* < 0.01, *** *p* < 0.001. Sessions/month refers to the mean number of AI-tool sessions per month in the final quarter of follow-up; Δ ROI confidence is the 12-month minus baseline score. Abbreviations: AI, artificial intelligence; AIELI, AI Economic Literacy Index; ROI, return on investment. mo, months; Δ, change; ρ, Spearman rank correlation coefficient.

**Table 7:** Cox proportional hazards models for time to first documented AI-tool use (n = 96; 62 events).

Covariate	Crude HR (95% CI)	*p*-Value	Adjusted HR (95% CI) ^a^	*p*-Value
High baseline AIELI (≥14 vs. <14)	3.25 (1.85–5.71)	<0.001	1.93 (0.98–3.77) †	0.055
Baseline AIELI, per 5 points	2.40 (1.72–3.36)	<0.001	1.54 (1.02–2.31)	0.040
Baseline ROI confidence, per point	1.38 (1.19–1.61)	<0.001	1.18 (1.00–1.40)	0.055
Baseline perceived barriers, per point	0.89 (0.82–0.96)	0.003	0.96 (0.87–1.06)	0.433
Prior formal AI training (yes vs. no)	2.46 (1.49–4.07)	<0.001	2.61 (1.43–4.79)	0.002
Private-sector centre (vs. public)	1.43 (0.87–2.35)	0.160	1.17 (0.66–2.06)	0.586
Physician (vs. non-physician)	1.43 (0.86–2.38)	0.167	1.89 (1.06–3.36)	0.030
Age, per 10 years	0.67 (0.52–0.88)	0.003	0.68 (0.51–0.91)	0.010

^a^ Adjusted model including the continuous AIELI (per 5 points), ROI confidence, perceived barriers, prior training, sector, profession and age: likelihood-ratio test *p* < 0.001; Harrell’s C-index 0.747; scaled Schoenfeld residual tests *p* ≥ 0.12 for all covariates. † The adjusted HR for the dichotomised exposure was estimated in a separate model replacing the continuous AIELI with the high/low indicator and the same covariates. Abbreviations: AI, artificial intelligence; AIELI, AI Economic Literacy Index; CI, confidence interval; HR, hazard ratio; ROI, return on investment. n, number of participants; C-index, concordance index.

**Table 8:** Covariate balance before and after inverse probability of treatment weighting, and effect of high baseline AIELI on sustained AI-tool use across analytical specifications.

Covariate/Specification	Unweighted (High vs. Low)	SMD Before	Weighted (High vs. Low)/Estimate (95% CI)	SMD After/*p*-Value
**Covariate balance (high vs. low AIELI)**				
Age, years	40.3 vs. 45.2	−0.50	41.8 vs. 42.0	−0.02
Female sex	70.2 vs. 56.4	0.29	68.1 vs. 66.3	0.04
Physician	45.6 vs. 23.1	0.49	35.3 vs. 26.2	0.20
Private-sector centre	45.6 vs. 46.2	−0.01	45.0 vs. 48.8	−0.08
Prior AI training	47.4 vs. 30.8	0.35	41.6 vs. 41.2	0.01
Digital literacy	7.1 vs. 6.5	0.39	6.9 vs. 6.9	0.02
Professional experience, years	13.7 vs. 18.4	−0.50	15.1 vs. 15.2	−0.02
Weekly patient contacts	41.2 vs. 40.7	0.03	41.8 vs. 41.6	0.01
**Effect estimates for sustained use (high vs. low AIELI)**				
Crude RR (complete case, n = 91)	2.97 (1.46–6.06)			0.003
Covariate-adjusted RR (modified Poisson)	2.25 (1.07–4.74)			0.033
IPTW-weighted RR (stabilised ATE weights)	2.24 (1.15–4.34)			0.018
Doubly robust RR (IPTW + covariate adjustment)	2.07 (1.04–4.12)			0.038
IPTW-weighted HR for time to first use	2.53 (1.47–4.35)			<0.001
Multiple imputation RR (20 imputations, n = 96)	2.89 (1.42–5.88)			0.003
Worst-case RR for loss to follow-up (n = 96)	2.48 (1.27–4.84)			0.003
Adjusted RR, alternative cut-off AIELI ≥15	1.68 (0.89–3.15)			0.107
Mantel–Haenszel RR stratified by profession (n = 91) ‡	2.80 (1.36–5.76)			0.005
Mantel–Haenszel RR, physicians and nurses only (n = 72) ‡	2.82 (1.23–6.46)			0.015
E-value for IPTW RR (point estimate; CI limit)	3.90; 1.57			—

Propensity-score model AUC 0.767; stabilised weights range 0.44–2.58 (mean 0.96), truncated at the 99th percentile (one weight). Continuous covariates are shown as means and binary covariates as percentages. RRs from modified Poisson regression with robust standard errors; the E-value is the minimum strength of association (risk-ratio scale) that an unmeasured confounder would need with both exposure and outcome to explain away the IPTW estimate. ‡ Post hoc analyses added during peer review; Mantel–Haenszel RRs with Greenland–Robins 95% CIs were computed from the profession strata in Table 4. Analysis populations and methods are summarised in Table A2. Covariate sets and coding for each adjusted and doubly robust model, the sample used to fit the propensity model, the application of the weights to each analysis population, propensity-score overlap and effective weighted sample sizes are given in Table A6. Abbreviations: AI, artificial intelligence; AIELI, AI Economic Literacy Index; ATE, average treatment effect; AUC, area under the receiver operating characteristic curve; CI, confidence interval; HR, hazard ratio; IPTW, inverse probability of treatment weighting; RR, risk ratio; SMD, standardised mean difference. n, number of participants.

**Table 9:** Exploratory decomposition of the association between high baseline AIELI and sustained AI-tool use into components through and not through 6-month ROI confidence (n = 88; 1000 bootstrap resamples).

Quantity	Estimate (95% CI)	*p*-Value
Path a: high AIELI → 6-month ROI confidence (mean difference, points)	2.06 (1.13 to 3.02)	<0.001
Path b: 6-month ROI confidence → sustained use (log-odds per point)	0.38 (0.08 to 0.95)	0.008
Exposure × mediator interaction (log-odds)	—	0.749
Total association (risk difference, high vs. low)	0.284 (0.052 to 0.473)	0.014
Direct association not through ROI confidence (risk difference)	0.147 (−0.083 to 0.365)	0.202
Indirect association via ROI confidence (risk difference)	0.137 (0.023 to 0.261)	0.020
Ratio of indirect to total association ^a^	48.2% (4.3 to 189.6)	—
Conditional indirect association: public hospital	0.182 (0.023 to 0.388)	0.016
Conditional indirect association: private centre	0.058 (−0.113 to 0.233)	0.390
Index of moderated mediation (private − public)	−0.125 (−0.383 to 0.106)	0.336

Mediator model: linear regression of 6-month ROI confidence on exposure and covariates; outcome model: logistic regression of sustained use on exposure, mediator and covariates (age, sector, profession, prior AI training, digital literacy). Effects are expressed as risk differences averaged over the covariate distribution, with Monte Carlo integration over the mediator; percentile bootstrap 95% CIs and two-sided bootstrap *p*-values. Conditional effects were estimated in sector-stratified models. ^a^ Not interpreted as the proportion of a causal effect transmitted through ROI confidence; the interval extends beyond 0–100%. Early use may be affected by economic literacy and may confound the association between 6-month ROI confidence and sustained use; the estimates do not identify a natural indirect effect. The assumed temporal structure is described in Section 2.4. Abbreviations: AI, artificial intelligence; AIELI, AI Economic Literacy Index; CI, confidence interval; ROI, return on investment. n, number of participants.

## Data Availability

The individual-level questionnaire and audit-log data are not publicly available because the ethics approval and the participant consent restrict their use to the study team and do not permit sharing of individual-level data. Aggregate data supporting the reported findings are presented in the article and in Appendix A.

## Abbreviations

The following abbreviations are used in this manuscript:

AI	Artificial intelligence
AIELI	AI Economic Literacy Index
AUC	Area under the receiver operating characteristic curve
CI	Confidence interval
HR	Hazard ratio
IPTW	Inverse probability of treatment weighting
IQR	Interquartile range
MD	Mean difference
OR	Odds ratio
RD	Risk difference
ROI	Return on investment
RR	Risk ratio
SD	Standard deviation
SMD	Standardised mean difference
STROBE	Strengthening the Reporting of Observational Studies in Epidemiology
VIF	Variance inflation factor

## Appendix A

Table A1 summarises enrolment, follow-up completion and sustained use by profession and centre; Table A2 summarises analysis populations and methods for Table 8; Table A3 gives the operational definitions of logged AI-tool use; Table A4 summarises the chronology of study documents and analytical specifications; Table A5 summarises the timing of the baseline assessment relative to first use and the analyses restricted to participants unused at assessment; Table A6 specifies the adjusted and doubly robust models in Table 8, including propensity-score overlap and effective sample sizes.

**Table A1:** Enrolment, follow-up completion and sustained AI-tool use by profession and centre.

Group	Enrolled, n	Completed, n (%)	Sustained Use, n/N (%)
Physician (medical/radiation oncologist)	35	34 (97.1)	17/34 (50.0)
Oncology nurse	40	38 (95.0)	13/38 (34.2)
Pharmacist/administrator	21	19 (90.5)	6/19 (31.6)
Public hospital	52	49 (94.2)	19/49 (38.8)
Private centre	44	42 (95.5)	17/42 (40.5)
All participants	96	91 (94.8)	36/91 (39.6)

All enrolled participants had an active institutional account with access to at least one AI tool. Completion percentages use the number enrolled in each group as the denominator; sustained-use percentages use completers. Counts are consistent with the profession and centre strata in Table 4. Overall, 62/96 participants (64.6%) had at least one documented AI-tool session during follow-up. Abbreviations: AI, artificial intelligence; n, number of participants; N, number of completers.

**Table A2:** Analysis populations and methods for the estimates in Table 8.

Estimate in Table 8	Analysis Population	Analysis Method
Crude RR	91 completers: 53 high, 38 low AIELI	Unweighted comparison of sustained-use risks.
Covariate-adjusted RR	91 completers	Modified Poisson regression with HC1 robust standard errors.
Adjusted RR, AIELI ≥15	91 completers	Adjusted analysis with an alternative literacy threshold of 15 points.
IPTW-weighted RR	91 completers	Stabilised ATE weights; robust standard errors.
Doubly robust RR	91 completers	IPTW combined with covariate adjustment.
IPTW-weighted HR	96 enrolled; 62 first-use events	Weighted time-to-first-use analysis; robust standard errors.
Multiple imputation RR	96 enrolled	Chained equations, 20 imputations; pooled using Rubin’s rules.
Worst-case RR	96 enrolled	Four high-literacy participants lost to follow-up assigned non-use; one low-literacy participant assigned sustained use: 29/57 versus 8/39.
Mantel–Haenszel RR by profession	91 completers	Stratified by physician, oncology nurse and pharmacist/administrator groups.
Mantel–Haenszel RR, physicians and nurses	72 completers	Stratified by physician and oncology nurse groups.

Propensity scores for high AIELI were estimated by logistic regression using age, sex, profession, sector, prior AI training, digital literacy, years of experience and weekly patient contacts. Stabilised weights targeted the average treatment effect and were truncated at the 99th percentile. Post hoc Mantel–Haenszel analyses used Greenland–Robins confidence intervals and the profession strata in Table 4. Covariate sets, coding, weight application and effective sample sizes are given in Table A6. Abbreviations: AIELI, AI Economic Literacy Index; ATE, average treatment effect; HC1, heteroskedasticity-consistent standard errors (type 1); HR, hazard ratio; IPTW, inverse probability of treatment weighting; RR, risk ratio.

**Table A3:** Operational definitions of logged AI-tool use.

Element	Definition (Counted as Use)	Recorded but Not Counted/Handling
Contouring module (version 2.4)	Generation of an automatic organ-at-risk contour set for a planning CT of a patient record (one inference request = one event).	Single sign-on log-in, opening of the module, viewing or export of previously generated contours: present in the audit log, not counted.
Documentation assistant (version 5.1; 5.2 from September 2025)	Generation of a structured treatment summary or coding suggestion for a patient record (one generation request = one event).	Log-in, page views, re-opening or printing of previously generated summaries: present in the audit log, not counted. The version update did not change the logged event types.
Prescription safety-check module (version 1.8)	User-initiated submission of a chemotherapy prescription for dose and interaction checking (one submission = one event). No automatic background checks were configured during follow-up.	Log-in, opening of the module, viewing of previous check reports: present in the audit log, not counted.
Log-ins and page views	—	Recorded by the single sign-on and audit systems; never counted as use.
Training and test interactions	—	Performed in a separate training tenant with fictitious patient identifiers; written to a separate training log that was not part of the production extracts. No manual exclusion of production records was performed.
Session	One or more qualifying events by the same user in the same tool with gaps of less than 30 min between consecutive events.	Activity in a second tool opened a separate session.
Active month	A 30-day period counted from the centre-specific roll-out date with at least one session in any tool.	Months 10–12 define the sustained-use window (active in at least two of the three).
Sessions per month, final quarter	Mean number of sessions per 30-day period over months 10–12, all tools combined.	—
First use	Timestamp of the first qualifying event in any tool.	Compared with the questionnaire-completion timestamp (Table A5).

Definitions were recorded in the protocol amendment (version 1.1, Section 3) before enrolment. Audit logs were extracted monthly from the production tenants of the three tools by an information-technology officer and merged with questionnaire data by the pseudonymised identifier. Abbreviations: AI, artificial intelligence; CT, computed tomography.

**Table A4:** Chronology of study documents and analytical specifications.

Document or Specification	Version and Date	Content Fixed at That Time
Study protocol	Version 1.0, 15 December 2023; ethics approval 30 January 2024 (code 044)	Two-centre cohort design, eligible population, 12-month follow-up, AIELI (0–25) as exposure, log-based primary outcome, questionnaire schedule, consent to linkage.
Protocol amendment	Version 1.1, 8 January 2025 (before enrolment)	Licenced tools and versions, centre-specific roll-out dates (6 and 13 January 2025), audit-log extraction procedure, tool-specific qualifying events (Table A3).
Baseline questionnaire and scoring key	Version 1.0, 20 December 2024; administered unchanged 13 January–14 March 2025	AIELI items and 0–25 scoring key, ROI confidence, perceived financial barriers, willingness to invest, covariates; electronic completion timestamp.
Statistical analysis plan	24 March 2026 (before final log extraction and linkage on 3 April 2026)	AIELI ≥14 dichotomy, alternative cut-off ≥15, four literacy bands, propensity-score covariates and IPTW, doubly robust model, imputation procedures, exploratory indirect-association analysis.
Post hoc analyses	Added during peer review, 2026	Mantel–Haenszel RRs stratified by profession; comparison of questionnaire-completion and first-use timestamps; delayed-entry and post-assessment time-origin Cox models; sustained-use analysis excluding prior users.

Specifications in the first three rows were contemporaneous with data collection; those in the last two rows were analytical choices made after enrolment. The protocol, its amendment and the baseline questionnaire are retained by the study team with their version histories and are available from the corresponding authors on reasonable request. Abbreviations: AIELI, AI Economic Literacy Index; IPTW, inverse probability of treatment weighting; ROI, return on investment; RR, risk ratio.

**Table A5:** Timing of the baseline assessment relative to first recorded use, and analyses restricted to participants unused at assessment.

Quantity	High AIELI (n = 57)	Low AIELI (n = 39)	All (n = 96)
Interval from centre roll-out to questionnaire completion, days, median (IQR)	22 (10–39)	27 (13–44)	24 (11–41)
Recorded use before questionnaire completion, n (%)	8 (14.0)	1 (2.6)	9 (9.4)
Interval from first use to questionnaire completion among prior users, days, median (range)	9 (2–31)	17	9 (2–31)
Participants unused at assessment, n	49	38	87
First-use events after assessment, n	37	16	53
Delayed-entry Cox model, crude HR (95% CI)	2.98 (1.62–5.48)	Reference	*p* < 0.001
Delayed-entry Cox model, adjusted HR (95% CI) ^a^	1.81 (0.89–3.69)	Reference	*p* = 0.102
Time origin at questionnaire completion, crude HR (95% CI)	2.91 (1.59–5.33)	Reference	*p* < 0.001
Sustained use among prior users, n/N	7/8	1/1	8/9
Sustained use excluding prior users, n/N (%)	22/45 (48.9)	6/37 (16.2)	28/82 (34.1)
Crude RR excluding prior users (95% CI)	3.01 (1.36–6.64)	Reference	*p* = 0.002
Covariate-adjusted RR excluding prior users (95% CI) ^b^	2.19 (0.99–4.86)	Reference	*p* = 0.054

Questionnaire-completion timestamps were taken from the electronic baseline form and first-use timestamps from the audit logs (Table A3). The delayed-entry model used time from centre-specific roll-out with entry into the risk set at questionnaire completion. ^a^ Adjusted for the covariates in Table 7 (ROI confidence, perceived barriers, prior training, sector, profession and age). ^b^ Modified Poisson regression with the covariate set in Table A6. Abbreviations: AIELI, AI Economic Literacy Index; CI, confidence interval; HR, hazard ratio; IQR, interquartile range; RR, risk ratio.

**Table A6:** Specification of the propensity-score model and of the adjusted and doubly robust models in Table 8.

Model	Sample	Covariates and Coding	Weights and Weighted Sample
Propensity-score model (logistic regression for high AIELI)	96 enrolled	Age (years, continuous); sex (female vs. male); profession (physician [reference], oncology nurse, pharmacist/administrator); sector (private vs. public); prior formal AI training (yes vs. no); self-rated digital literacy (1–10, continuous); professional experience (years, continuous); weekly patient contacts (continuous). AUC 0.767.	Stabilised ATE weights = P(A)/PS for the high group and P(1 − A)/(1 − PS) for the low group. PS range 0.26–0.93 (high) and 0.08–0.87 (low); common support 0.26–0.87 with 87/96 inside. Weights 0.44–2.58 (mean 0.96) after truncation at the 99th percentile (one weight, 3.12 → 2.58).
Covariate-adjusted RR (modified Poisson, HC1)	91 completers	High vs. low AIELI plus age (per 10 years), sex, profession (three-level), sector, prior AI training, digital literacy (per point) and professional experience (per 10 years).	Unweighted.
Adjusted RR, cut-off ≥15	91 completers	As above, with the exposure defined as AIELI ≥15 vs. <15.	Unweighted.
IPTW-weighted RR (modified Poisson, robust SE)	91 completers	Exposure only.	Weights estimated in the 96 enrolled participants and applied without re-estimation; ESS 80.3 (47.0 high, 30.7 low).
Doubly robust RR (IPTW + covariate adjustment)	91 completers	Exposure plus the full covariate set of the covariate-adjusted RR; profession entered as a three-level factor to address the residual physician imbalance after weighting (SMD 0.20).	Same weights; ESS 80.3.
IPTW-weighted HR for time to first use (Cox, robust SE)	96 enrolled; 62 events	Exposure only; time from centre-specific roll-out.	Weights as estimated; ESS 84.6 (50.2 high, 31.9 low).
Multiple imputation RR	96 enrolled	Imputation model: sustained use, exposure, all propensity-score covariates, baseline ROI confidence and perceived barriers (chained equations, 20 imputations); analysis model: crude RR pooled by Rubin’s rules.	Unweighted.
Delayed-entry Cox models (Table A5)	87 unused at assessment; 53 events	Crude; adjusted for Table 7 covariate set.	Unweighted.

ESS, effective sample size calculated as (Σw)^2^/Σw^2^ from the stabilised weights (Kish). Weighted SMDs in Table 8 were calculated in the 96 enrolled participants. Abbreviations: AI, artificial intelligence; AIELI, AI Economic Literacy Index; ATE, average treatment effect; AUC, area under the receiver operating characteristic curve; ESS, effective sample size; HC1, heteroskedasticity-consistent standard errors (type 1); HR, hazard ratio; IPTW, inverse probability of treatment weighting; PS, propensity score; ROI, return on investment; RR, risk ratio; SE, standard error; SMD, standardised mean difference.
